# Nanocurcumin is superior to native curcumin in preventing degenerative changes in Experimental Cerebral Malaria

**DOI:** 10.1038/s41598-017-10672-9

**Published:** 2017-08-30

**Authors:** Chaitanya Dende, Jairam Meena, Perumal Nagarajan, Viswanathan Arun Nagaraj, Amulya Kumar Panda, Govindarajan Padmanaban

**Affiliations:** 1 Department of Biochemistry, Indian Institute of Science, Bengaluru, 560012 India; 2 National Institute of Immunology, New Delhi, 110067 India; 3 Infectious Disease Biology, Institute of Life Sciences, Bhubaneswar, 751023 India

## Abstract

Curcumin has many pharmacological activities despite its poor bioavailability and *in vivo* stability. Here, we show that a nanoformulated curcumin (PLGA-curcumin) has better therapeutic index than native curcumin in preventing the onset of neurological symptoms and delaying the death of mice in experimental cerebral malaria. Oral PLGA-curcumin was at least as effective as native curcumin at a 15-fold lower concentration in preventing the breakdown of blood-brain barrier and inhibition of brain mRNAs for inflammatory cytokines, chemokine receptor CXCR3 and its ligand CXCL10, with an increase in the anti-inflammatory cytokine IL-10. This was also reflected in serum cytokine and chemokine levels. At equivalent concentrations, a single oral dose of PLGA-curcumin was more effective in inhibiting serum IFNγ levels and enhancing IL-10 levels than native curcumin. Even at low concentrations, PLGA-curcumin was superior to native curcumin in inhibiting the sequestration of parasitized-RBCs and CD8^+^ T cells in the brain. A single oral dose of 5 mg PLGA-curcumin containing 350 μg of curcumin resulted in 3–4 fold higher concentration and prolonged presence of curcumin in the brain than that obtained with 5 mg of native curcumin, indicating better bioavailability of PLGA-curcumin. PLGA-curcumin has potential as an adjunct drug to treat human cerebral malaria.

## Introduction

Curcumin (diferuloylmethane) from turmeric plant *Curcuma longa* and its derivatives known as curcuminoids have been recognized to be effective in experimental studies for a very large number of diseases with an equally large number of diverse molecular targets^1, 2^. More specifically, curcumin has been tested as an anti-inflammatory agent for treatment of cancers and other diseases^3, 4^. Based on its immunomodulatory properties, curcumin has also been suggested as an adjunct drug for the treatment of many infectious diseases^5^. In spite of extensive clinical trials using curcumin, and its classification as a generally recognized as safe (GRAS) molecule, it is still not an approved drug even for a single ailment^6^. The main reasons include poor water solubility, low bioavailability, rapid metabolism and clearance from body^6, 7^. It has been suggested that nanocurcumin represents a promising therapeutic advancement over native curcumin^8^. It is in this context that we want to report our results obtained with curcumin nanoparticles prepared using poly(lactide-co-glycolide) (PLGA) in comparison with native curcumin to treat experimental cerebral malaria (ECM).

Human cerebral malaria (HCM) is a serious complication with high mortality (15–25%) despite the existing standard therapy using artemisinin derivatives. Hence, there is a need for adjunct therapy^9^. Inflammation in brain, blood-brain barrier (BBB) breakdown and sequestration of parasitized-RBCs (pRBCs) are the major factors contributing to high mortality in HCM. Despite some differences, ECM in *Plasmodium berghei*-infected mice has been shown to share many features of HCM and is considered very useful to dissect the mechanisms involved and screen drug molecules^10–12^. Curcumin, when given orally by gavage during 0–5 days post-infection, has been shown to delay death of mice (C57BL/6) in the ECM model, reversing the symptoms of cerebral malaria^13^. We have recently shown that native curcumin alone is effective in counteracting the neurological symptoms and inflammation parameters of ECM with a significant delay in the mortality of mice, which eventually died due to anemia caused by parasite build-up in blood^14^.

The objective of the present study was to formulate curcumin in low molecular weight PLGA-based nanoparticles (PLGA-curcumin) and evaluate its potential in ECM. PLGA polymers have been extensively used for drug and vaccine delivery^15, 16^. PLGA- and PLA-based polymers provide interesting features for the sustained delivery of curcumin while protecting its degradation and improving its bioavailability^17–19^. In a fresh set of experiments carried out in parallel, we compared the efficacies of orally administered native curcumin and PLGA-curcumin. It was observed that PLGA-curcumin was as effective as native curcumin at a 15-fold lower concentration in counteracting the neurological symptoms and inflammation parameters, and in preventing the breakdown of BBB. It was more effective than native curcumin in preventing the sequestration of lymphocytes and pRBCs in brain. PLGA-curcumin, even at a lower curcumin concentration, led to higher concentrations of curcumin in the brain than native curcumin. The results indicated that PLGA-curcumin nanoparticles have the potential to be used as adjunct therapy for treating HCM.

## Results

### Preparation and characterization of PLGA-curcumin

PLGA nanoparticles entrapping curcumin were prepared using single emulsion solvent evaporation method. Use of acetone and dichloromethane for solubilization of curcumin and PLGA resulted in stable emulsion formation leading to formulation of spherical nanoparticles. The size and zeta potential of curcumin nanoparticles were 495 ± 14.4 nm and −13.8 ± 1.8 Mv, respectively, as measured by Nano zetasizer. Curcumin nanoparticles were found to be spherical and non-aggregated as analyzed by transmission electron microscopy. Size of the PLGA nanoparticles obtained by TEM was in accordance with the results obtained by zetasizer (Fig. S1a). PLGA-curcumin nanoparticles showed narrow size distribution and polydispersity index (PDI = 0.24). *In vitro* release of curcumin form PLGA nanoparticles showed characteristic burst release followed by sustained release for 12 days (Fig. S1b). Around 15–20% of the total entrapped curcumin was released within six hours. Continuous release of curcumin was observed from the particles and around 65% was released in seven days. This *in vitro* release profile showed that low molecular weight PLGA (with inherent viscosity of 0.2 dl/gm) is suitable for sustained release of entrapped curcumin. Curcumin load and entrapment efficiency in PLGA nanoparticles were 70 μg/mg and 37.91%, respectively (Table S1).

### Effect on parasite growth

**Figure 1:**
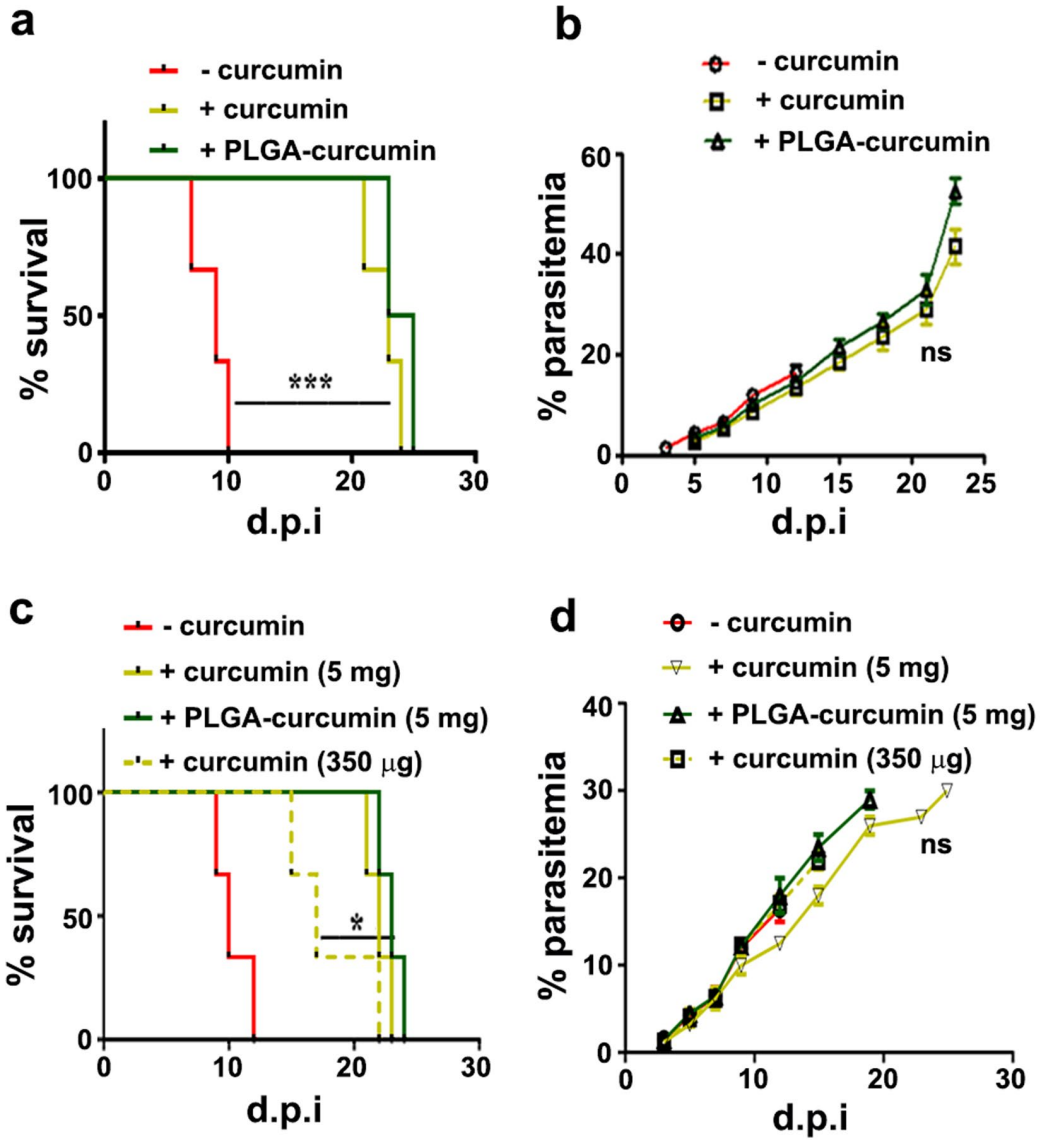
Effect of native curcumin and PLGA-curcumin on survival of *P. berghei*-infected mice and parasitemia in blood. (**a**,**c**) Percentage mortality of *P. berghei*-infected mice. (**b**,**d**) Percentage parasitemia of *P. berghei*-infected mice. Data are presented as mean + S.D. *P < 0.05; **P < 0.001; ***P < 0.0001; ns, not significant. The bar represents the partners compared.

The ECM studies were carried out in C57BL/6 mice infected with *P. berghei* ANKA strain. Around 80–90% of infected mice showed typical neurological symptoms between 7 to 10 days, eventually resulting in coma and death. Native curcumin/PLGA-curcumin was fed orally after three days post infection (d.p.i) and 5 mg of each was given in three oral doses at 24 h interval. The treatments were started before the onset of neurological symptoms. Since PLGA-curcumin contained 70 μg curcumin/mg, it provided only 350 μg of curcumin/dose. The results presented in Fig. 1a indicate that the infected mice without treatment died around 10–12 d.p.i. In accordance with results obtained in earlier studies^20, 21^, these mice showed typical neurological symptoms and died when the parasitemia was around 15% (Fig. 1b). At the concentrations provided, both the preparations could completely eliminate the neurological symptoms and the mice died of anemia between 22 to 25 d.p.i. (Fig. 1a), when the parasitemia was around 40% (Fig. 1b). Since PLGA-curcumin provided only 350 μg of curcumin/dose, it was of interest to compare the efficacy of native curcumin at 350 μg/dose with PLGA-curcumin. The results presented in Fig. 1c indicate that with three oral doses of native curcumin at 350 μg/dose, 75% of the mice died between 16–18 days, whereas with PLGA-curcumin, all the mice died between 22–25 days, similar to the results obtained with three oral doses of 5 mg native curcumin/dose. Some of them in the native curcumin group (350 μg) died by manifesting neurological symptoms with a parasitemia around 18–20% (Fig. 1d), whereas none of the mice given three doses of PLGA-curcumin or native curcumin (5 mg) showed neurological symptoms. In the rest of the experiments, the results obtained with three oral doses of 5 mg of native curcumin were compared with three oral doses of 5 mg PLGA-curcumin, providing 350 μg of native curcumin/dose. Data from PLGA alone controls were very similar to those obtained from infected animals without drug treatment.

### Effect on BBB breakdown and pRBC sequestration in brain

**Figure 2:**
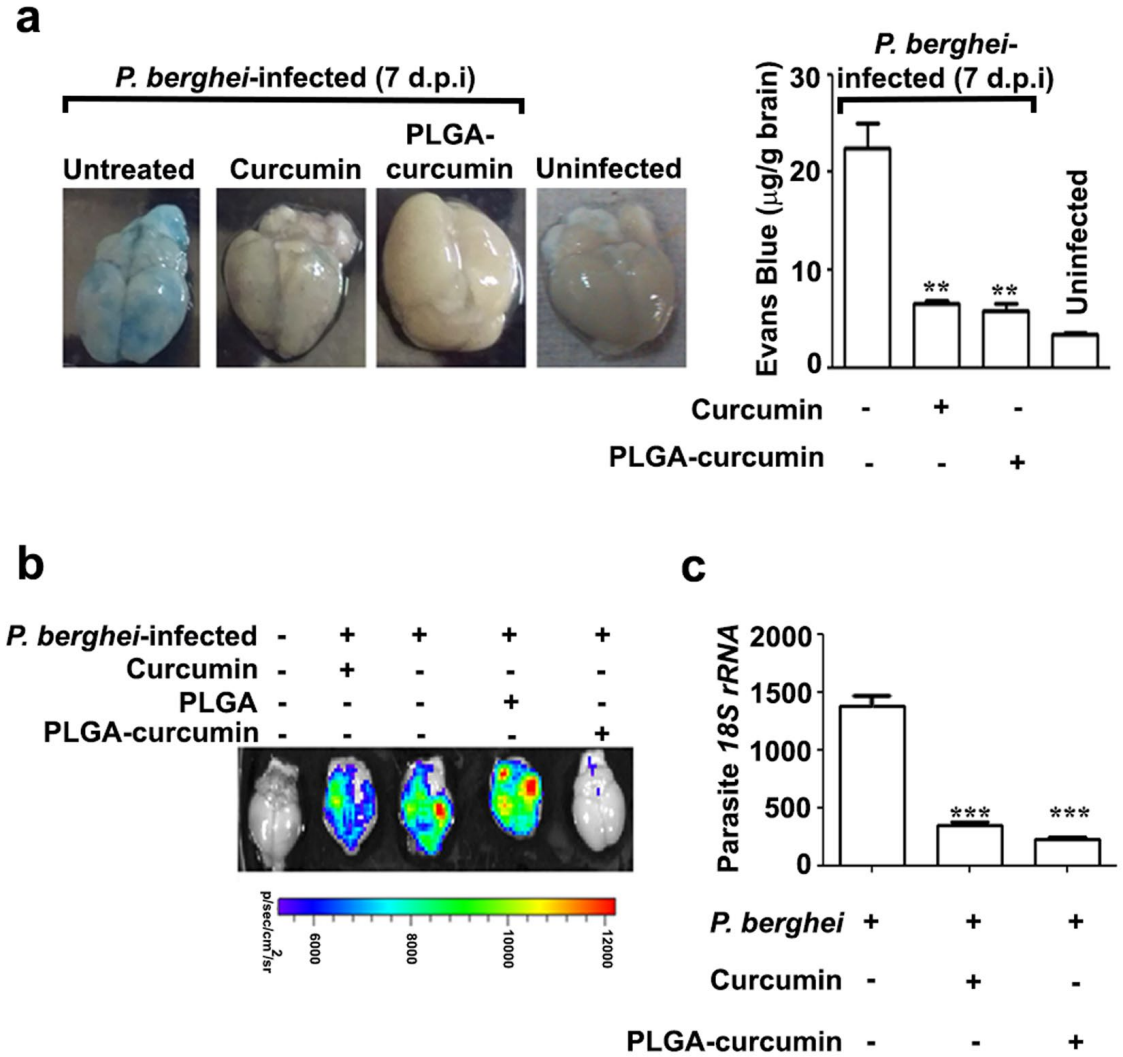
Comparison of the effects of native curcumin and PLGA-curcumin on BBB breakdown and pRBC sequestration in *P. berghei*-infected mice. (**a**) Evans Blue staining of brain in *P. berghei*-infected mice. (**b**) *Ex vivo* bioluminescence imaging of brain in *P. berghei*-infected mice. (**c**) Quantitative PCR for parasite *18S rRNA* in the brain samples of *P. berghei*-infected mice. P value comparison is between infected and treated animals.

A crucial event in HCM and ECM is the breakdown of the BBB associated with the sequestration of pRBCs and lymphocytes in the brain^12, 22, 23^. We have shown earlier that native curcumin can counteract this derangement^14^, measured in terms of Evans Blue staining of the brain. The results presented in Fig. 2a indicate that PLGA-curcumin was at least as effective as native curcumin in preventing the breakdown of BBB. Next, pRBC sequestration in the brain was measured using *P. berghei* ANKA strain expressing luciferase. The mice were injected with luciferin and the bioluminescence of the brain was measured *ex vivo*. The results presented in Fig. 2b indicate that PLGA-curcumin was superior to native curcumin in inhibiting pRBC sequestration. While native curcumin treatment showed a low level of pRBC sequestration, brain from PLGA-curcumin treated mice could not be distinguished from uninfected mice. PLGA particles alone used as control did not prevent pRBC sequestration in the brain. Whole body imaging carried out on day 5, 7 and 10 post infection and the quantification of bioluminescence indicated that PLGA-curcumin was more effective than native curcumin in preventing pRBC sequestration (Fig. S2). Parasite concentration in the brain was also assessed by performing quantitative PCR for parasite *18S rRNA*. This also showed greater inhibition with PLGA-curcumin than that obtained with native curcumin. (Fig. 2c).

### Effect on inflammatory parameters

**Figure 3:**
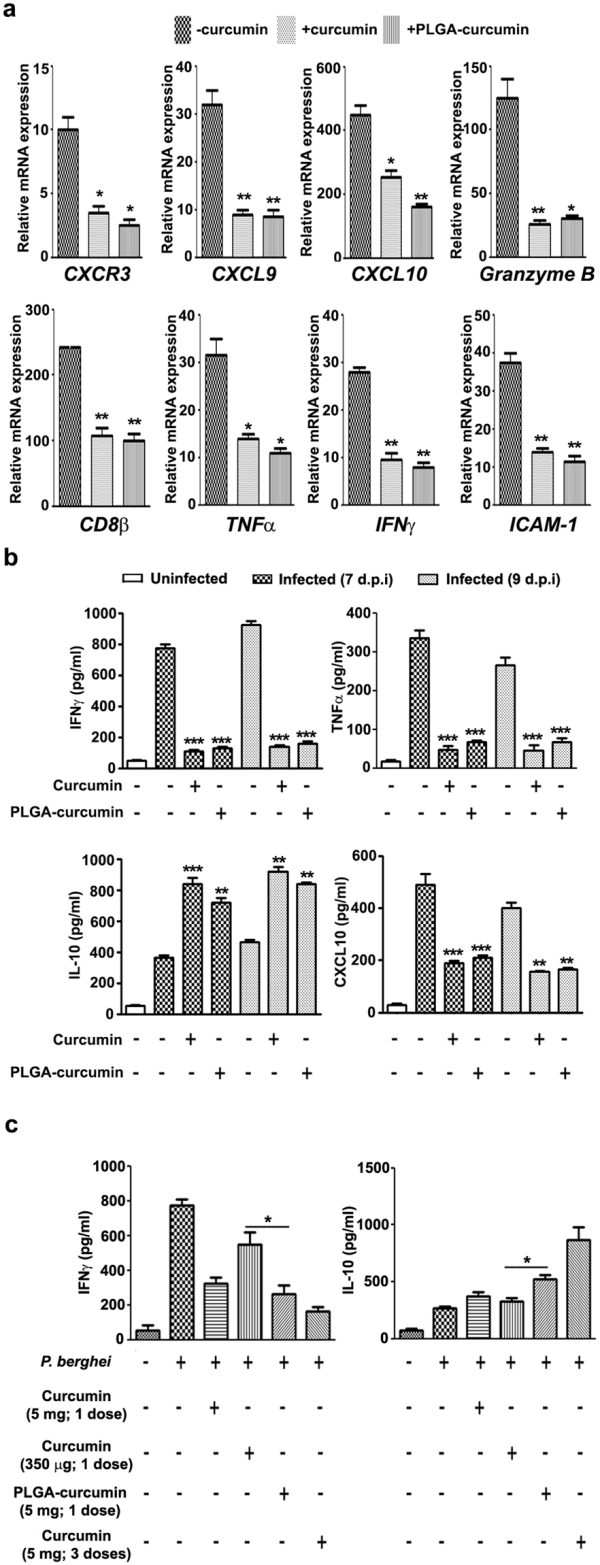
Comparison of the effects of native and PLGA-curcumin on cytokine and chemokine levels. (**a**) Relative mRNA expression in the brain samples of *P. berghei*-infected mice. (**b**,**c**) Inflammatory and anti-inflammatory cytokine and chemokine levels in the serum samples of *P. berghei*-infected mice. Comparison is between infected and treated animals. Bar represents the partners compared.

ECM is characterized by a striking increase in inflammatory cytokines and chemokines associated with the breakdown of BBB and lymphocyte sequestration^24–26^. The results presented in Fig. 3a indicate that both native curcumin and PLGA-curcumin were effective in counteracting the increase in brain mRNAs for chemokines (CXCL9 and CXCL10), chemokine receptor (CXCR3), inflammatory cytokines (IFNγ and TNFα), inflammatory marker (Granzyme B), CD8^+^ T cell marker (CD8β) and adhesion molecule (ICAM-1). The inflammatory (IFNγ and TNFα) and anti-inflammatory cytokine (IL-10) and chemokine (CXCL10) levels were measured in serum as well. The results presented in Fig. 3b indicate that IFNγ, TNFα, CXCL10 and IL-10 levels increased in the infected serum. Native curcumin and PLGA-curcumin inhibited the striking increase in the serum levels of the inflammatory cytokines and the chemokine. Interestingly, both native curcumin and PLGA-curcumin further enhanced the levels of the anti-inflammatory cytokine (IL-10) in serum. The difference between native curcumin and PLGA-curcumin in terms of efficacy was clearly seen with single dose data, providing 350 μg curcumin in either case. The results indicate that the increase in IFNγ was inhibited by 25% and 75% with native curcumin and PLGA-curcumin treatments, respectively. There was a marginal further increase in IL-10 levels with native curcumin and a 100% increase with PLGA-curcumin (Fig. 3c).

### Effect on NF-kB phosphorylation

**Figure 4:**
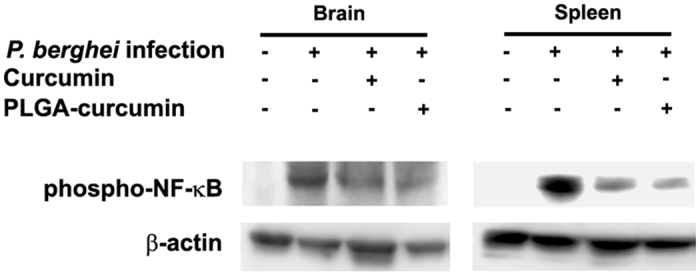
Comparison of the effects of native curcumin and PLGA-curcumin given as per the standard dose schedule on phosphorylation of NF-κB. The images of whole blots are given in Figure S3.

NF-κB activation is an upstream event in enhancing the transcription of inflammatory markers including cytokines^27^. Since p65 phosphorylation is a key step in the activation process, the levels of phospho-p65 were measured in the brain lysates. Both native curcumin and PLGA-curcumin treatments led to a decrease in the levels of phospho-P65. A similar pattern was also observed in spleen lysates (Fig. 4).

### Effect on sequestration of CD8^+^ T cells in the brain using FACS analysis

**Figure 5:**
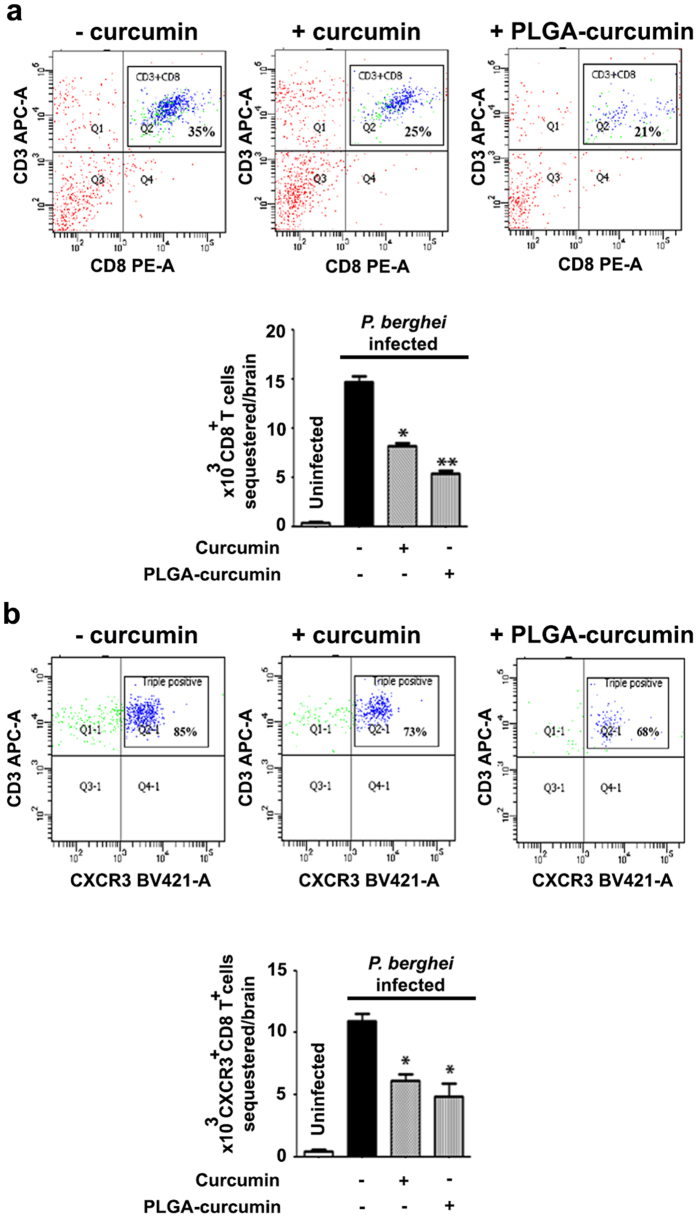
Comparison of the effects of native curcumin and PLGA-curcumin given as per the standard dose schedule on CD8^+^ T cell sequestration in the brain of *P. berghei*-infected mice. (**a**,**b**) FACS analysis of lymphocyte population in the brain samples of *P. berghei*-infected mice for CD3^+^/CD8^+^ (double positive) and CD3^+^/CD8^+^/CXCR3^+^ (triple positive) cells. Bar represents comparison between infected and curcumin/PLGA-curcumin treated animals.

FACS analysis of lymphocyte population in the brain was carried out for CD3^+^/CD8^+^ (double positive) and CD3^+^/CD8^+^/CXCR3^+^ cells (triple positive). CXCR3 and its ligands are reported to be essential for precipitating ECM and CXCR3-deficient lymphocytes are 7-fold less efficient than wild type cells for migration into the infected brain^25^. The results presented in Fig. 5a and b indicate that native curcumin and PLGA-curcumin inhibited sequestration of the CD8^+^ T cells. Native curcumin and PLGA-curcumin treatments inhibited double positive cells by 50% and 60%, respectively and triple positive cells by 40% and 60%, respectively.

### Bioavailability of curcumin

**Figure 6:**
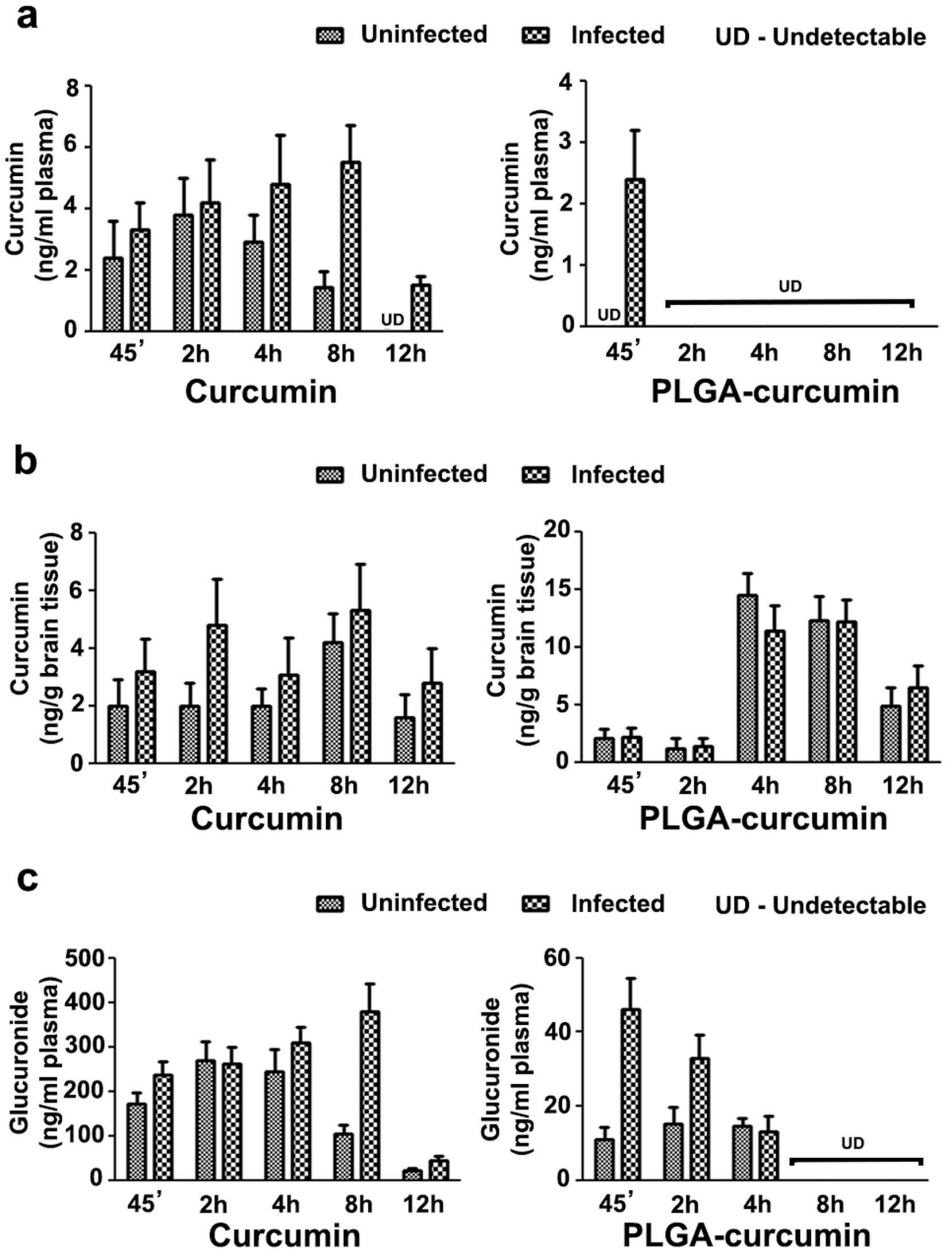
Estimation of curcumin and its glucuronide in plasma and brain of *P. berghei*-infected and uninfected mice. (**a**) Curcumin concentration in plasma samples. (**b**) Curcumin concentration in brain samples. (**c**) Glucuronide derivative concentration in plasma samples.

A single oral dose of 5 mg of native curcumin or PLGA-curcumin (providing 350 μg of curcumin) was given to mice and the levels of free curcumin and the glucuronide derivative were measured in plasma and brain by LC/MS analysis (Fig. 6). With curcumin treatment, the levels of free curcumin were in the range of 2–5 ng/ml of plasma or g of brain between 45 min and 8 h. It was below the limit of detection after 12 h. The glucuronide concentration ranged between 170 to 370 ng/ml in plasma between 45 min and 8 h, indicating rapid metabolism of curcumin. Interesting results were obtained with PLGA-curcumin treatment. 1–2 ng of curcumin/ml was detected in plasma up to 2 h and undetectable after this period. In the brain, the same pattern was seen up to 2 h, but the concentration increased to 12–14 ng/g after 4 h. The concentration was still around 5 ng/g after 12 h. The glucuronide concentration was around 10 to 40 ng/ml in the plasma and this correlated with the fact that PLGA-curcumin provided only 350 μg of curcumin. In general, plasma and brain showed a higher concentration of curcumin in infected mice than the uninfected controls. Thus, the concentration of curcumin in brain after a single dose of oral PLGA-curcumin (350 μg of curcumin) was significantly higher than that derived from native curcumin (5 mg).

## Discussion

Various formulations of turmeric and curcumin in milk and natural lipids have been described in ancient literature for the treatment of a variety of ailments^1, 2^. More recent efforts are in terms of using milk proteins and phospholipids to make curcumin nanoparticles to improve stability, bioavailability and efficacy. One such example is the use of β-casein micelle as a nanocarrier to improve the solubility of curcumin. It has been shown that β-casein from camel could increase the curcumin solubility by at least 2500-fold^28^. Curcumin liposomes have been prepared using milk fat globule membrane phospholipids exhibiting superior properties in terms of encapsulation efficiency, smaller particle size, slower *in vitro* release etc., compared to native curcumin^29^. In another study, curcumin has been encapsulated in milk exosomes and shown to have higher stability in PBS, sustain a harsher digestive process and manifest ability to cross the intestinal barrier than free curcumin^30^. A recent study describes the effect of five different formulations of curcumin in milk and indicates higher statistically significant prevention of inflammation in two arthritic rat models. The study also concludes that addition of adjuvants like ghee can enhance the bioavailability of curcumin^31^.

Such curcumin formulations using natural products need to be evaluated in detail against chemical-based formulations, including nanoformulations, that are reported to enhance bioavailability of curcumin in plasma and improve efficacy mainly in different experimental cancer studies^32–34^. Among these, PLGA-curcumin is a preferred option, since PLGA polymer is FDA approved for delivery of drugs and functional foods in view of its biodegradability and biocompatibility^16, 17^. One of the advantages of polymer particle based delivery system is that, when administered orally, it protects the entrapped curcumin from gastric and enzymatic degradation, and hydrolysis. Based on studies with PLGA-curcumin nanoparticles in the rat model, it has been suggested that the increased oral bioavailability may be due to improved water solubility, higher rate of release in the intestinal juice, increased permeability, inhibition of P-glycoprotein-mediated efflux pump and increased residence time in the jejunum^35^. While most studies report on the efficacy of PLGA-curcumin in different disease models and cell lines, data on curcumin uptake after administration to animals *in vivo* are scarce. In particular, data on curcumin concentration in the brain when given orally are not available. When PLGA-curcumin was administered intravenously in the rat, curcumin was detected in different organs including the brain, indicating that it can cross the BBB. Nanoformulation was shown to significantly increase the retention time in the cerebral cortex and hippocampus, with curcumin concentrated mainly in the latter site^36^.

In the present study, the results establish that orally administered curcumin (native as well as PLGA-curcumin) was able to cross the BBB. An interesting feature with PLGA-curcumin treatment was the enhanced and delayed uptake of curcumin in the brain. The small peak (1–2 ng) seen at 45 min was possibly due to the un-entrapped curcumin present. Unlike native curcumin, PLGA-curcumin treatment resulted in a slower build-up of curcumin concentration in the brain giving rise to 3–4 fold increase in the peak after 4 h. PLGA-curcumin maintained the concentration of around 5 ng/g for more than 12 h in brain. It needs to be pointed out that this was seen with 5 mg PLGA-curcumin providing only 350 μg curcumin compared to 5 mg of native curcumin. The lower concentration of curcumin in plasma in case of PLGA-curcumin as compared to native curcumin was because of the lower amount of curcumin release in the initial four hours and rapid metabolism. Once the nanoparticles reached the brain, continuous and enhanced hydrolysis and degradation of low molecular weight polymer and slower metabolism of curcumin in brain as compared to plasma could lead to a slower build-up of curcumin concentration. The use of low molecular weight PLGA for entrapment of curcumin is ideal for slow and sustained build-up of curcumin concentration in brain for more than 12 h. It has been shown that nanoparticles below 200 nm size can cross the tight junction of BBB and can deliver the drugs into the CNS. Even though the size of PLGA nanoformulations used in this study was more than 480 nm, it still showed higher concentration of curcumin in brain when compared to native curcumin. This could be due to the rupture of BBB in case of cerebral malaria infection and use of 1% mannitol as resuspending and cryoprotectant agent during PLGA nanoformulations. This could have helped the PLGA nanoformulation to pass the tight junction and cross the BBB.

In terms of therapeutic efficacy, the results obtained in the present study establish for the first time the potential of PLGA-curcumin in the treatment of ECM. The results indicate that PLGA-curcumin was at least as effective as native curcumin in terms of counteracting the breakdown of BBB and reversing all the molecular parameters governing the inflammatory responses seen in ECM. This greater efficacy was seen at 350 µg of curcumin/dose provided by 5 mg of PLGA-curcumin compared to native curcumin provided at 5 mg/dose. Even at this lower concentration PLGA curcumin showed greater efficacy in preventing the sequestration of pRBCs and CD8^+^ T cells in the brain and reducing the levels of IFNγ and IL-10 in serum. However, further studies would be needed to optimize the dose and schedule of PLGA-curcumin administration. It would also be of interest to prepare PLGA-curcumin of different sizes and curcumin load for evaluation. Ghosh *et al*.^37^ have prepared nanotized curcumin using a modified emulsion diffusion-evaporation method without the use of polymer matrices and shown that the preparation, when given as a biweekly dose for a month, was more effective at an equivalent concentration than native curcumin in delaying the onset of neurological symptoms and death in mice. The nanotized preparation was also shown to yield high bioavailability by measuring total curcumin in plasma that included the glucuronide and sulfate derivatives. But, data on curcumin levels in the brain and mechanistic details in terms of counteraction of the breach in BBB and sequestration of pRBCs as well as CD8^+^ T cells are not available. In our study, three doses of PLGA-curcumin were shown to completely prevent the onset of neurological symptoms and the delayed death was due to anemia. The results obtained in our study and also reviewed elsewhere^6, 7^ indicate that curcumin is metabolized very fast to its glucuronide derivative, with free curcumin accounting for a very small fraction of the estimate. Therefore, it would be of interest to measure free curcumin levels in plasma as well as brain with nanotized curcumin and investigate whether the nanotized curcumin can only delay the onset of neurological symptoms in mice as stated by the authors or it eliminates the symptoms completely.

In terms of the mechanism of action of curcumin, it needs to be pointed out that most of the studies in literature^1, 8^ would suggest that inhibition of a variety of molecular targets *in vitro* in cell lines are seen only with concentrations of curcumin in excess of 20 μM. In the present study, curcumin concentration was found to be in the range of 4–15 ng/ml of plasma or g of brain, obtained with a single dose of native or PLGA-curcumin. These concentrations would approximately translate to 10–40 nM. We have also earlier demonstrated the long-term effect of native curcumin in preventing parasite recrudescence in mice^38^. We have proposed that curcumin, perhaps, acts through potentiating the mechanisms involved in immune memory to parasite antigens^5, 38^. Such a mechanism can explain the efficacy of curcumin to bring about changes long after it has disappeared from circulation, be it counteraction of parasite recrudescence in an experimental uncomplicated malaria model or prevent the degenerative neurological changes in the ECM model^39^. At the same time, even at nanomolar levels, a 3–4 fold increase in curcumin concentration brought about by PLGA-curcumin elicited a significantly higher potential to counteract the neurological effects seen in ECM than that obtained with native curcumin. Further studies would be needed to understand the unique mechanism of curcumin action.

There are a couple of examples where nanocurcumin formulations have been taken further for human studies. One is a commercial preparation referred to as theracurcumin, which contains a highly absorptive form of curcumin with small particle size. This has been shown to decrease serum atherosclerotic low density lipoprotein levels in chronic obstructive pulmonary disease (COPD) patients, an inflammatory disease caused essentially by smoking^40^. In another study, Meriva, a commercial curcumin-phosphotidyl choline complex has been shown to significantly improve clinical and biochemical end points in a study with 100 osteoarthritic patients. The standard clinical end point scores were complemented with effect on inflammatory markers such as IL-1β, IL-6, soluble CD40 ligand, soluble vascular cell adhesion molecule-1, and erythrocyte sedimentation rate^41^. Thus, the data obtained in our study on ECM with PLGA-curcumin warrant it to be taken forward for evaluation as a potential adjunct therapy to treat HCM.

## Materials and Methods

### Chemicals

PLGA 50:50, 20 kDa ester terminated, was purchased from Purac, Holland. Curcumin, PVA (30–70 kDa), MTT solution, DMSO cell culture grade and mannitol were purchased from Sigma-Aldrich, USA. Dichloromethane (DCM), acetone and methanol were purchased from Merck India. Uranyl acetate solution and DMEM were purchased from Life technologies, USA, and copper grids were purchased from Polysciences, Warrington, USA. All other chemicals used were of high purity grade available commercially.

### PLGA-curcumin nanoparticles preparation

PLGA-curcumin nanoparticles (PLGA 50:50, 20 kDa) were prepared by single emulsion solvent evaporation method with slight modifications^42^. Briefly 300 mg of PLGA polymer and 60 mg of curcumin were dissolved in 5 ml of organic phase (3 ml dichloromethane (DCM) + 2 ml acetone) for 4 h and volume was made up to 5 ml using DCM prior to nanoparticles formation. The organic phase containing curcumin and PLGA was emulsified in 15 ml aqueous phase containing 1% PVA (30–70 kDa) as emulsifier with drop by drop addition and sonication at 40% Watt 20 duty cycle for 3 min (Branson sonifier USA, HD 2200, MS-72 probe) to form O/W emulsion. Similarly, dummy PLGA nanoparticle was prepared without curcumin in organic phase. O/W emulsion formed was subjected to magnetic stirring (150 rpm) for 7 h to ensure complete DCM evaporation at room temperature. After complete evaporation of organic phase, the nanosuspension was centrifuged at 16000 rpm for 15 min. Nanoparticles pellet was washed twice with milli-Q water and resuspended in 5 ml of 1% mannitol solution, lyophilized and stored at 4 °C for further use.

### PLGA-curcumin nanoparticle characterization

Curcumin nanoparticles were characterized for particle size, zeta potential and polydispersity using Malvern Zetasizer UK (Nano ZS). Briefly, 1 mg of nanoparticles was resuspended in 1 ml milli-Q water and suitably diluted with milli-Q water to avoid multi-scattering. The nanoparticle suspension was vortexed and particle size, zeta potential and polydispersity were measured using nano ZS analyzer in three different batches and the results were expressed as mean + SD.

### Transmission Electron Microscopy (TEM)

Morphology of PLGA-curcumin nanoparticles was analyzed using transmission electron microscope (TEM). Nanoparticle formulations were suitably diluted with milli-Q water, two drops of nanosuspension were placed on copper grid (excess water removed with filter paper) and dried at room temperature for 90 sec. Sample was negatively stained with 1% (w/v) uranyl acetate solution for 30 seconds, dried and observed using TEM (CM 10, Phillips, Holland), operating at 80 kV. Images were obtained using digital imaging software AMT image capture engine (version 5.42.391).

### Curcumin load and entrapment efficiency

Load and entrapment efficiency of curcumin in curcumin nanoparticles was determined by UV visible spectrophotometer. Briefly 10 mg of curcumin nanoparticles was dissolved in 1 ml of DCM and left for 4 h for complete dissolution of PLGA polymer. After 4 h, DCM was evaporated to 200 μl at 37 °C followed by 1 ml methanol addition to precipitate the PLGA polymer. Curcumin was extracted into methanol by centrifugation at 13000 rpm for 10 min at 4 ^◦^C. This process of curcumin extraction by DCM dissolution and methanol extraction was repeated till all the curcumin was extracted. The methanol fractions were pooled and DCM was evaporated at 37 ^◦^C. Curcumin concentration in the extracted methanol was assayed by UV visible spectrophotometer at 420 nm by using the standard graph of curcumin in methanol in the linear range of 5–60 μg/ml. Curcumin load was determined as follows: Load = Curcumin in nanoparticles/total nanoparticles; Entrapment efficiency was determined as follows:

$${\rm{Entrapment}}\,{\rm{efficiency}}\,( \% )=({\rm{Theoretical}}\,\mathrm{load}/\mathrm{Practical}\,{\rm{load}})\times {\rm{100}}{\rm{.}}$$

### *In vitro* curcumin release study

*In vitro* curcumin release study was performed with PLGA-curcumin nanoparticles to know the release. Briefly, 20 mg of curcumin nanoparticles were suspended in 20 ml phosphate buffer saline and methanol mixture (PBS: methanol, 9:1 pH 7.4,) (For triplicate 60 mg in 60 ml PBS: methanol) and aliquots were made in 1.7 ml centrifuge tube in triplicates and kept on rotator shaker at 37 °C and 100 rpm. Samples were collected after centrifugation of the nanosuspension at 16000 rpm for 15 min at predetermined time intervals. After complete sample collection, curcumin concentration in the collected samples were determined with the UV visible spectrophotometer using the standard graph of curcumin prepared in the PBS and methanol mixture (PBS: methanol, 9:1 pH 7.4,) at linear range of 1–60 µg/ml. Release study was performed in triplicate on the same nanoformulation batch. Results were expressed as mean + SD.

### Ethics statement

Animals were maintained in accordance with the norms of Committee for the Purpose of Control and Supervision of Experimental Animals (CPCSEA), Government of India (Registration No: 48/1999/CPCSEA) and as approved by the Institutional Animal Ethics Committee (IAEC) of the Indian Institute of Science, Bangalore (CAF/Ethics/102/2007-435 and CAF/ Ethics/192/2010).

### ECM in mice and curcumin/ PLGA-curcumin treatment

C57BL/6 mice, 7 to 8 weeks old, were used for the purpose. *P. berghei* ANKA strain (MRA-311, MR4, ATCC Manassas Virginia) was maintained in these mice and 3 × 10^6^ parasitized red blood cells (pRBCs) were injected as described^13^. Around 80–90% of the mice manifested neurological symptoms typical of ECM between 7 and 10 days. The animals ended up with coma and in general, died between 10–12 d.p.i. Native curcumin or PLGA-curcumin was given orally in three doses at 24 h interval, starting day 3 post infection. Native curcumin was given in DMSO (5 mg/dose) and PLGA-curcumin as water suspension (5 mg/dose, providing 350 μg of curcumin). The assay procedures carried out were essentially as described earlier^13^.

### Blood-brain barrier permeability and *in vivo* imaging

Evans Blue dye was used to assess blood-brain barrier. 0.2 ml of the dye (2% in PBS) was given by i.v. and the staining of the brain was assessed after 2 h. A quantitative measurement was also obtained by extracting the dye from the brain with 100% formamide and the absorbance was measured at 620 nm^12^.

### Whole body and *ex vivo* imaging to assess the sequestration of pRBCs in the brain

For this purpose, MRA-796 plasmid expressing GFP-luciferase obtained from MR4 (BEI Resources Depository, NIAID, NIH) was transfected into *P. berghei* ANKA strain to obtain a stable transgenic line of *P. berghei*, expressing GFP-luciferase. C57BL/6 was infected with this transgenic *P. berghei* ANKA line (3 × 10^6^ pRBC). D-luciferin (Promega, VivoGloTM) was injected on the days mentioned and bioluminescence of the whole body and head was measured after 10 min. *Ex vivo* imaging of the isolated brain was also carried out as described^13, 43^. The head and whole body imaging was performed with medium binning factor and field of view (FOV) as 4 cm and 21.8 cm. The exposure time was between 50 –300 sec based on the intensity of the signal. For *ex vivo* imaging of the brain, the animals were sacrificed and the brain was isolated after whole body perfusion. Average radiance (p/sec/cm2/sr) values were calculated for region of interest (ROI) using Living Image® 4.3.1(64 bit) software for experimental and control animals.

### qPCR analysis for markers of inflammation

Real Time PCR was carried out with 1 µg of RNA isolated from brain (7 d.p.i.) using TRI Reagent (Sigma-Aldrich). ABI StepOnePlusTM System was used. Random hexamers were used for reverse transcription and the cDNA was diluted 1:25 times to carry out Real Time PCR. Housekeeping gene *18s rRNA* was used as endogenous control. The following transcripts were quantified: CXCR3, CXCL9, CXCL10, ICAM-1, TNFα, IFNγ, granzyme B and parasite *18s rRNA*. Uninfected animal CT values served as experimental controls. The relative gene expression levels were calculated by 2^−ΔΔCT^ method. The primers used are listed in Table S2.

### Cytokine and chemokine analysis in serum

ELISA was carried out for the measurement of the inflammatory cytokines IFNγ and TNFα, anti-inflammatory cytokine IL-10 and the chemokine ligand CXCL10 in serum. Murine ELISA kits from BD OptEIATM were used to measure IFNγ and IL-10 levels in 20 µl serum as per manufacturer’s protocol. TNFα and CXCL10 levels were quantified using Krishgen Biosystems ELISA kits. Biotinylated detection coupled Avidin-HRP antibodies were used to generate a colored product using 3′,3′,5′,5′ tetramethylbenzidine (TNB) as substrate. The reaction was stopped using 2 N H2SO4. The colored product was measured at A450 using the Infinite 200 PRO TECAN microplate reader. The data provided represent mean + SD from 3 animals in each group.

### Phospho NF-κB estimation

Western blot analysis was carried out on 7 d.p.i. brain and spleen lysates. Whole organ lysate was obtained by homogenizing brain/spleen in 0.1 M potassium phosphate buffer containing 0.25 M sucrose and centrifuging the homogenate at 14000 g for 20 min. The supernatant (50 μg protein) was used for SDS-PAGE analysis. Western blot analysis was carried out with p-NF-κB antibody (1:1000, CST Rabbit monoclonal # 3033) and anti-rabbit HRP conjugate. The blots were developed using ECL substrate (Millipore) with ImageQuant LAS 4000 machine.

### FACS analysis of leukocytes sequestered in the brain

Tissue sequestered leukocytes were isolated and processed as described earlier^43^. Briefly, brain from uninfected and infected mice (7 d.p.i), were used for the purpose. Brains were harvested from different groups of animals after cardiac puncture and whole body perfusion. Brains were maintained at 4 ^◦^C in the serum containing DMEM media until digestion. Each brain was cut into pieces and digested with 0.05% collagenase D (Roche) and 2U/ml of DNaseI (Thermo scientific) at 37 ^◦^C for 20 min. The tissue extract was sieved through nylon mesh (70 μm) and centrifuged at 400x*g* for 5 min. The pellet was resuspended in DMEM medium and layered on 30% (v/v) percoll (Sigma Aldrich) and centrifuged at 400x*g* for 20 min. The cell suspension was treated with RBC lysis buffer (155 mM NH4Cl, 10 mM NaHCO3, 0.1 mM EDTA, pH 7.3) to remove RBCs. Cell viability was checked using Trypan blue (Sigma Aldrich) and 10^7^ cells were used for flow cytometry. Cell surface staining was performed using conjugated CD3e (clone145-2C11, BD Biosciences), CXCR3/CD183 (clone CXCR3-173, eBioscience), CD8a antibodies (clone53-6.7, BD Biosciences) for 1 h at 4 ^◦^C. Cells were washed with DMEM and fixed with 4% paraformaldehyde followed by PBS wash. Cells were finally suspended in PBS and used for FACS analysis in BD FACS Canto™ II machine. Results were analyzed using BD FACS DIVA™ software.

### Estimation of curcuminoids in plasma and brain

Curcumin estimation was carried out as described earlier^13^. C57BL/6 mice were given a single oral dose of curcumin or PLGA-curcumin (5 mg/mouse). Plasma or brain samples, fresh or frozen, were processed for curcumin extraction. SPE cartridge (Strata-X 33 μ polymeric reversed phase; 8B-S100-UBJPhenomenex) was used in the case of plasma samples to enrich the phenolics. An LC system (Waters, Corporation, Milford, U.S.A) consisting of an Acquity ultra Performance LC and electrospray chemical ionization tandem mass spectrometer (ESCI-MS/MS; Waters) was used. The samples were separated on Ambient BEH C18 (2.1 × 50 mm), 1.7 μ or Equivalent L1 column. The LOQ for curcumin was 10 ppb. The plasma and tissue sample residue were reconstituted with an appropriate volume of acetonitrile: water containing 0.1% formic acid (1:1) and transferred into a micro-vial. A 5 μl aliquot was injected into LC-MS/MS system and analyzed for curcuminoids and metabolites. Data acquisition and quantitation were performed using MassLynx software version 4.1. These analyses were carried out at Arjuna Natural Extracts, Cochin, India. The samples were coded and analyzed in a blinded fashion.

### Statistical analysis

Paired or grouped statistical tests were carried out according to the sample size. One-way analysis of variance (Bonferroni’s Multiple Comparison Test) or Unpaired two tailed t tests were performed using GraphPad Prism 5. Data are presented as mean + S.D. P value summary is mentioned on the bar of each figure. *P < 0.05; **P < 0.001; ***P < 0.0001. ns, not significant. Standard survival curve analysis was performed using Kaplan-Meier plot and P values calculated by Log-rank (Mantel-Cox) test.

### Data availability

All data generated or analysed during this study are included in this published article (and its Supplementary Information files).

## Electronic supplementary material

Supplementary Information
