# Supplementary material for: Nanocurcumin is superior to native curcumin in preventing degenerative changes in Experimental Cerebral Malaria
Source: Sci Rep. 2017 Aug 30;7:10062. doi: 10.1038/s41598-017-10672-9 (PMC5577147; doi:10.1038/s41598-017-10672-9)
Supplement: Supplementary file 1 — Supplementary Information [file 41598_2017_10672_MOESM1_ESM.pdf]

Supplementary Information

Nanocurcumin is superior to native curcumin in preventing degenerative changes in Experimental Cerebral Malaria

Chaitanya Dende[1], Jairam Meena[2], Perumal Nagarajan[2], Viswanathan Arun Nagaraj[3], Amulya Kumar Panda[2*] and Govindarajan Padmanaban[1*]

[1]Department of Biochemistry, Indian Institute of Science, Bengaluru - 560012, India.

[2]National Institute of Immunology, New Delhi - 110067, India.

[3]Infectious Disease Biology, Institute of Life Sciences, Bhubaneswar - 751023, India.

[*]amulya@nii.ac.in (A.K.P.); geepee@biochem.iisc.ernet.in (G.P.)

Supplementary Table S1. (C) PLGA-curcumin nanoparticles size, load and entrapment efficiency.

Formulation	Size (nm)	Zeta potential (mV)	PDI	Load (µg/mg)	E E (%)
PLGA dummy nanoparticles	382.4 ± 12.6	-11.2 ± 2.1	0.26	-	-
PLGA-curcumin nanoparticles	495 ± 14.4	-13.8 ± 1.8	0.24	65	37.91

Supplementary Table S2. Primers used in qPCR reactions.

Gene	*Gene sequence*
CXCR3	*Forward 5'AATGCCACCCATTGCCAGTAC 3'*<br>*Reverse 5' AGCAGTAGGCCATGACCAGAAG 3'*
CXCL9	*Forward 5' GCCATGAAGTCCGCTGTTCT 3'*<br>*Reverse 5' GGGTTCCTCGAACTCCACACT*
CXCL10	*Forward 5' GACGGTCCGCTGCAACTG 3'*<br>*Reverse 5' GCTTCCCTATGGCCCTCATT 3'*
TNFα	*Forward 5' AAGCCTGTAGCCCACGTCGTA3'*<br>*Reverse 5' GGCACCACTAGTTGGTTGTCTTTG 3'*
IFNγ	*Forward 5' TCAAGTGGCATAGATGTGGAAGAA*<br>*Reverse 5' TGGCTCTGCAGGATTTTCATG 3'*
ICAM-1	*Forward 5' GCCTCCGGACTTTCGATCTT 3'*<br>*Reverse 5' GTCAGGGGTGTCGAGCTTTG 3'*
CD8β	*Forward* 5' GCTGGTAGTCTGCATCCTGCTTC 3'<br>*Reverse* 5' TTGCTAGCAGGCTATCAGTCTTGTG 3'
18S rRNA P. berghei ANKA	*Forward 5' AAGCATTAAATAAAGCGAATACATCCTTAC 3'*<br>*Reverse 5' GGAGATTGGTTTTGACGTTTATGTG 3'*
Granzyme B	*Forward 5' CCTCCTGCTACTGCTGAC 3'*<br>*Reverse 5' GTCAGCACAAAGTCCTCTC 3'*

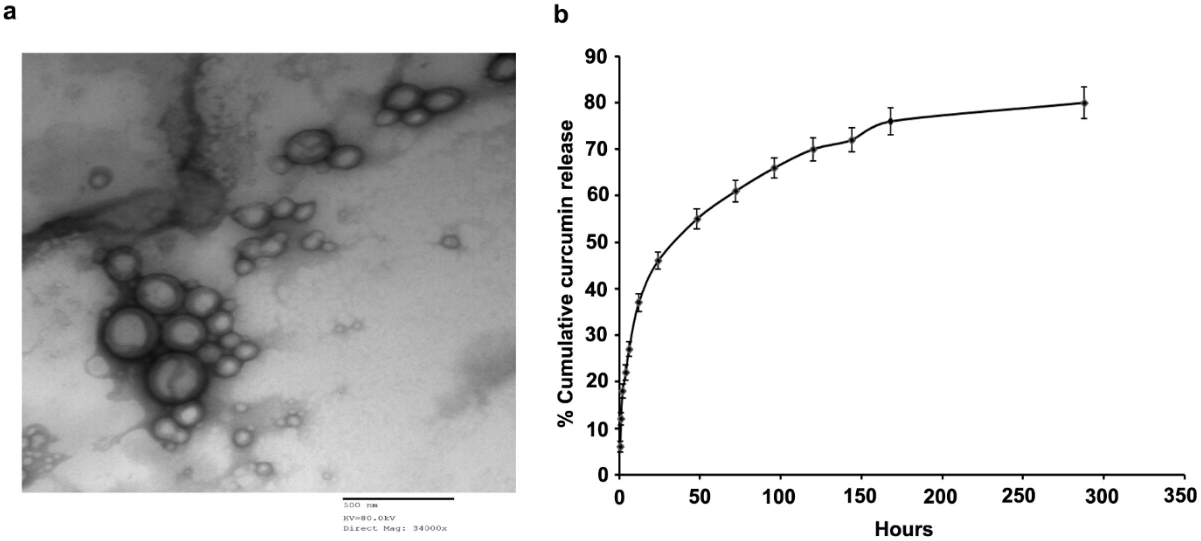

Figure S1. Characterization of PLGA-curcumin nanoparticles (**a**) TEM images of PLGA nanoparticles entrapping curcumin. The average size of particle is around 260 nm. (**b**) *In vitro* cumulative release of curcumin from PLGA nanoparticles.

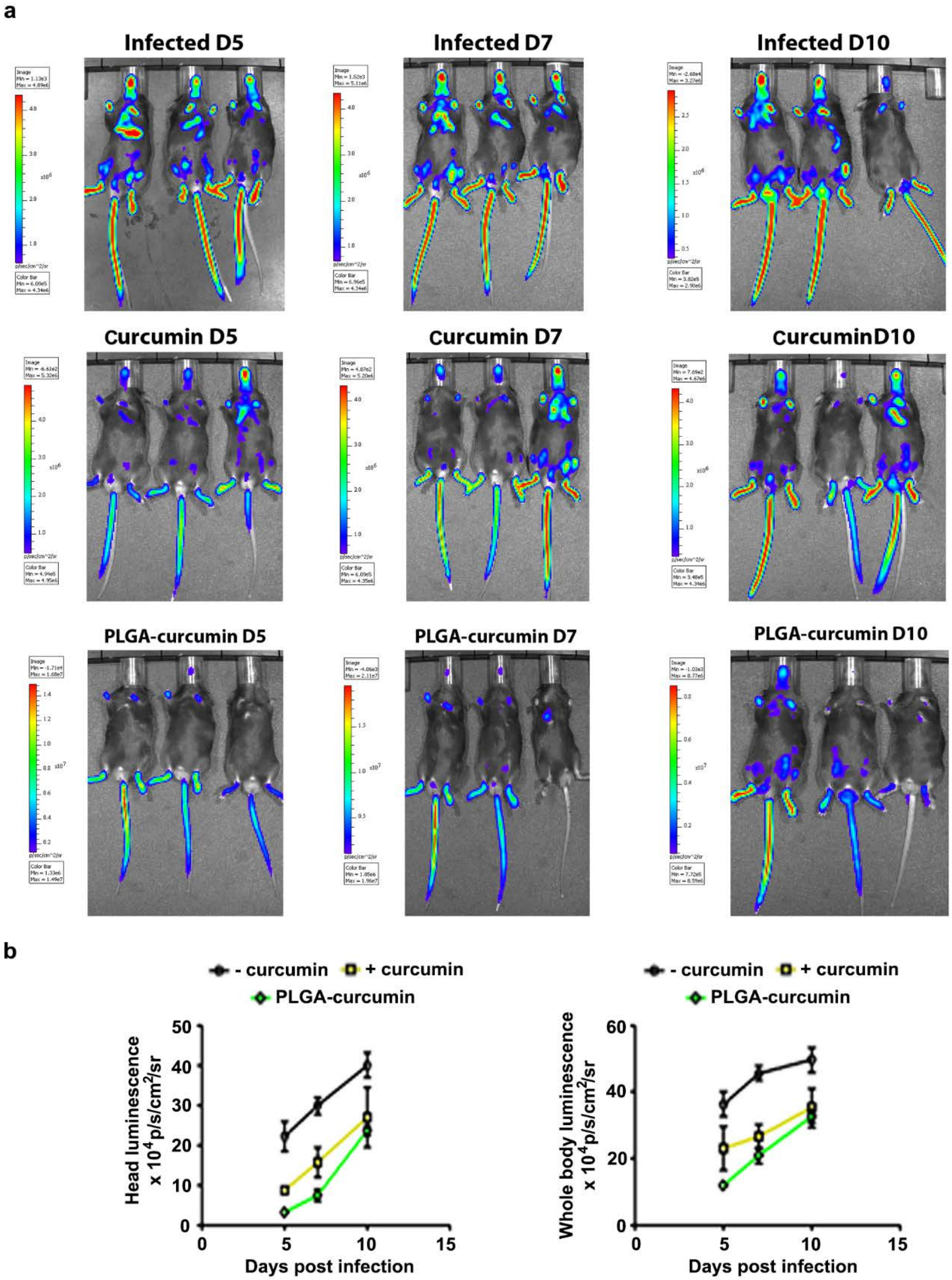

Figure S2. Quantification of bioluminescence. (**a**) Whole body bioluminescent images of untreated (- curcumin), native curcumin (+ curcumin) and PLGA-curcumin treated mice infected with *P. berghei*-ANKA transgenic parasites expressing luciferase. (**b**) Quantification of head and

whole body luminescence. The bioluminescence images were taken for three animals on days 5, 7 and 10. The animals were given three oral doses of native curcumin (5 mg) or PLGA-curcumin (5 mg, providing 350 μg of curcumin), starting on 3 d.p.i. The data given in the top two rows were published earlier[13] and are included here for comparison. Native curcumin and PLGA-curcumin data were generated in the same experiment.

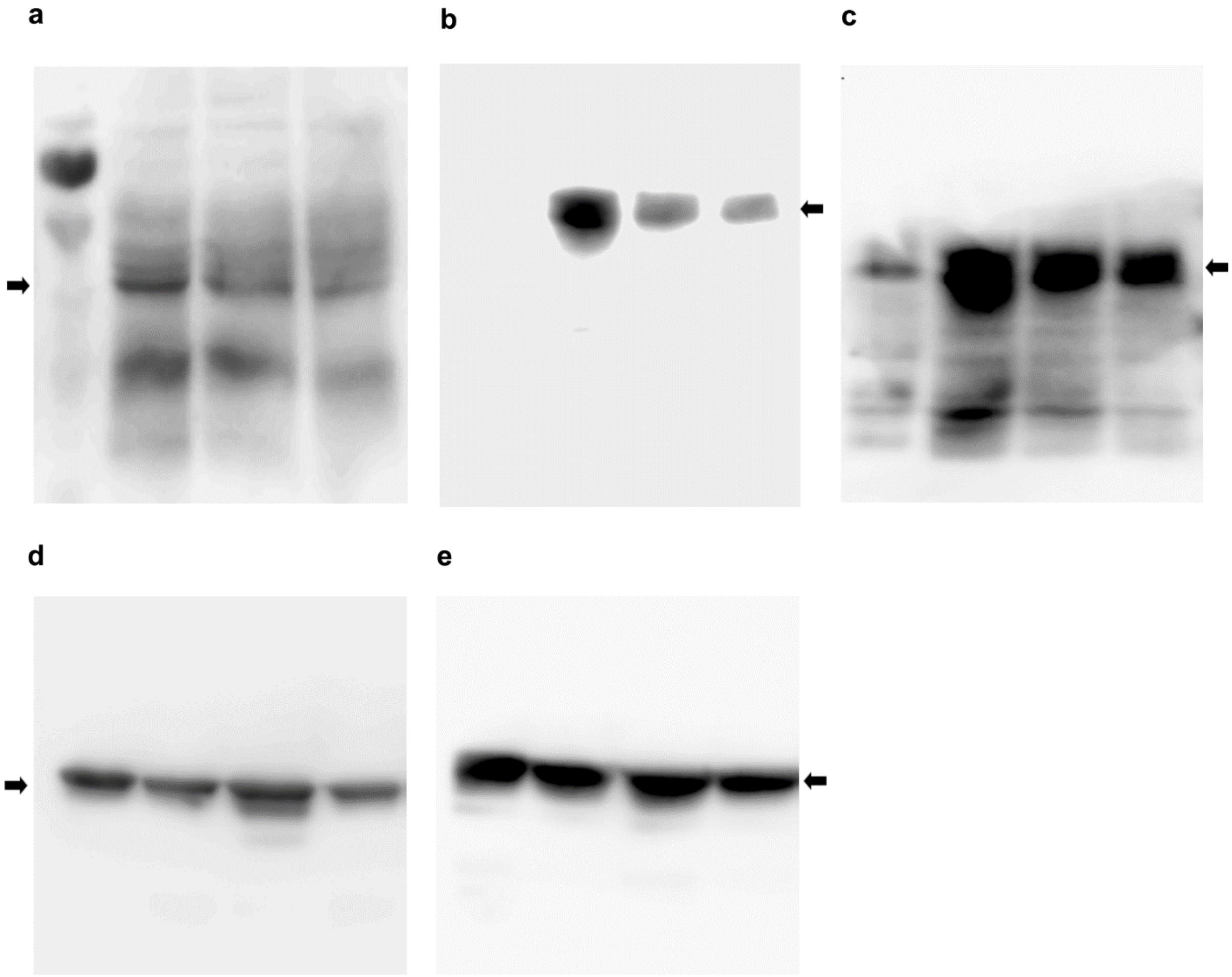

Figure S3. Whole blot images of Figure 4. (**a**) Whole blot image of brain phospho-NF-κB. (**b**) Whole blot image of spleen phospho-NF-κB (short exposure). (**c**) Whole blot image of spleen phospho-NF-κB (long exposure). (**d**) Whole blot image of brain β-actin used as control. (**e**) Whole blot image of spleen β-actin used as control. Arrows represent the protein bands given in Figure 4.
